# 5-HT Obesity Medication Efficacy via POMC Activation is Maintained During Aging

**DOI:** 10.1210/en.2014-1223

**Published:** 2014-07-22

**Authors:** Luke K. Burke, Barbora Doslikova, Giuseppe D'Agostino, Alastair S. Garfield, Gala Farooq, Denis Burdakov, Malcolm J. Low, Marcelo Rubinstein, Mark L. Evans, Brian Billups, Lora K. Heisler

**Affiliations:** Department of Pharmacology (L.K.B., B.D., G.D., A.S.G., G.F., D.B., B.B., L.K.H.) and Wellcome Trust/Medical Research Council Institute of Metabolic Science (M.L.E.), University of Cambridge, Cambridge, CB2 0QQ, United Kingdom; Rowett Institute of Nutrition and Health (G.D., L.K.H.), University of Aberdeen, Aberdeen, AB21 9SB, United Kingdom; Department of Molecular and Integrative Physiology (M.J.L., M.R.), University of Michigan Medical School, Ann Arbor, Michigan 48105; and Instituto de Investigaciones en Ingeniería Genética y Biología Molecular (M.R.), Consejo Nacional de Investigaciones Científicas y Técnicas, 1428 Buenos Aires, Argentina

## Abstract

The phenomenon commonly described as the middle-age spread is the result of elevated adiposity accumulation throughout adulthood until late middle-age. It is a clinical imperative to gain a greater understanding of the underpinnings of age-dependent obesity and, in turn, how these mechanisms may impact the efficacy of obesity treatments. In particular, both obesity and aging are associated with rewiring of a principal brain pathway modulating energy homeostasis, promoting reduced activity of satiety pro-opiomelanocortin (POMC) neurons within the arcuate nucleus of the hypothalamus (ARC). Using a selective ARC-deficient POMC mouse line, here we report that former obesity medications augmenting endogenous 5-hydroxytryptamine (5-HT) activity d-fenfluramine and sibutramine require ARC POMC neurons to elicit therapeutic appetite-suppressive effects. We next investigated whether age-related diminished ARC POMC activity therefore impacts the potency of 5-HT obesity pharmacotherapies, lorcaserin, d-fenfluramine, and sibutramine and report that all compounds reduced food intake to a comparable extent in both chow-fed young lean (3–5 months old) and middle-aged obese (12–14 months old) male and female mice. We provide a mechanism through which 5-HT anorectic potency is maintained with age, via preserved 5-HT–POMC appetitive anatomical machinery. Specifically, the abundance and signaling of the primary 5-HT receptor influencing appetite via POMC activation, the 5-HT_2C_R, is not perturbed with age. These data reveal that although 5-HT obesity medications require ARC POMC neurons to achieve appetitive effects, the anorectic efficacy is maintained with aging, findings of clinical significance to the global aging obese population.

Adiposity commonly accumulates with age from early adulthood (20 years old in humans) through late middle-age (65 years old in humans). This increase in adiposity has significant health implications; age-related obesity represents the primary cause of metabolic syndromes (1). Understanding the mechanisms underpinning age-associated adiposity has clinical implications both for obesity treatment and the prevention of chronic obesity-related comorbidities in the aging population (2).

To date, medications targeting 5-hydroxytryptamine (5-HT; serotonin) pathways have been among the most clinically successful obesity treatments (3). These include d-fenfluramine, which stimulates 5-HT release/blocks 5-HT reuptake, and sibutramine, which blocks 5-HT/noradrenaline (NA) reuptake. However, both compounds have been withdrawn from clinical use due to off-target effects (3, 4). Transgenic technology has aided in the identification of 5-HT receptors (5-HTRs) responsible for the therapeutic effect of 5-HT obesity medications; an effect principally mediated via G_q_-coupled 5-HT_2C_Rs (3). The therapeutic potential of selective activation of 5-HT_2C_Rs for obesity treatment was realized in the summer of 2012 with the U.S. Food and Drug Administration approval of 5-HT_2C_R agonist lorcaserin (Arena Pharmaceuticals) and its release in 2013.

Over the last decade, a primary mechanism through which 5-HT action, via 5-HT_2C_R activation, achieves effects on appetite and body weight has been revealed. Specifically, d-fenfluramine and 5-HT_2C_R agonists chiefly elicit appetitive effects through activation of pro-opiomelanocortin (POMC) neurons (5–10). Brain POMC, a critical mediator of energy balance, is abundantly expressed in the arcuate nucleus of the hypothalamus (ARC), and a smaller population is localized in the nucleus of the solitary tract (11, 12). Although the 5-HT_2C_R–POMC axis represents a critical target in the clinical treatment of obesity, recent reports demonstrate that endogenous ARC POMC regulation of energy balance is impaired with aging. In middle-aged mice (12 months old, comparable to a human 40 years old), ARC POMC neurons display an age-dependent upregulation of mammalian target of rapamycin (mTOR) signaling, which functions to reduce POMC neuronal activity, an effect reversed by mTOR inhibitor rapamycin (13). Furthermore, genetic ablation of endogenous inhibitors of mTOR promoted aged characteristics in ARC POMC neurons of young mice (14, 15). Middle-aged mice also display increased inhibitory agouti-related protein drive onto ARC POMC neurons, which functionally reduced POMC firing rate and decreased resting membrane potential compared with younger mice (16).

Given the importance of POMC in mediating 5-HT's appetitive effect, we investigated whether age-associated perturbed ARC POMC activity thereby reduces 5-HT compound anorectic potency.

## Materials and Methods

### Animals

Mice on a C57BL/6 background expressing enhanced green fluorescent protein (EGFP) under the control of POMC regulatory elements (POMC-EGFP mice) (8, 17), ARC-specific POMC-knockout mice (POMC^fneo/fneo^) (12), and wild-type littermates were maintained on a 12-hour light, 12-hour dark cycle (lights on at 7:00 am) with ad libitum access to chow diet and water unless otherwise stated. All experiments were in accordance with the United Kingdom Animals (Scientific Procedures) Act 1986.

### Body composition

Body composition was compared in young adult (3.2 months ± 9 days, n = 7) and middle-aged adult (13.1 months ± 11 days, n = 9) male C57BL/6 mice (Charles River, UK) using dual-energy x-ray absorptiometry Lunar PIXImus2 mouse densitometer (General Electric Medical Systems).

### Feeding studies

POMC^fneo/fneo^ and wild-type littermates (males: 3.4 months ± 7 days, POMC^fneo/fneo^ 47.3 ± 2.1 g and wild-type 30.1 ± 1.3 g, n = 12; females: 3.1 months ± 8 days, POMC^fneo/fneo^ 37.9 ± 1.5 g and wild-type 23.1 ± 1.8 g, n = 14) and POMC-EGFP young adult (males: 3.7 months ± 10 days, 28.1 ± 0.7 g, n = 8; females: 3.4 months ± 14 days, 22.0 ± 0.3 g, n = 6), and middle-aged adult (males: 13.1 month ± 20 days, 34.3 ± 0.7 g, n = 9; females: 13.4 months ± 12 days, 32.3 ± 0.6 g, n = 8) mice were individually housed for 1 week before experimentation and received a habituation ip injection of vehicle 1 day before experimentation. Mice were then treated with vehicle, lorcaserin (8 mg/kg), d-fenfluramine (3 mg/kg), or sibutramine (5 mg/kg) 45 minutes before the onset of the dark cycle (lights on at 7:00 am and off at 7:00 pm) and food was removed. At the onset of the dark cycle, chow pellets were replaced and 3- to 4-hour food intake measured. These studies were performed using a within-subjects experimental design, with a minimum of a 3-day treatment-free period.

### In situ hybridization histochemistry

Young adult (males: 3.2 months ± 9 days, n = 7; females: 3.2 months ± 3 days, n = 7) and middle-aged adult (males: 13.1 month ± 11 days, n = 5; females: 13.3 months ± 5 days, n = 7) C57BL/6 mice were anesthetized with pentobarbitone (50 mg/kg ip) and transcardially perfused with diethylpyrocarbonate-treated PBS followed by 10% neutral buffered formalin (Sigma). Brains were extracted, immersed in the same fixative overnight, cryoprotected in 20% sucrose for 24 to 48 hours and then sectioned coronally on a freezing sliding microtome at 25 μm. Tissue was collected in 5 equal series. In situ hybridization histochemistry was conducted using antisense radiolabeled riboprobes specific to the mRNA sequences of *ht2cr* (5-HT_2C_R) and *Pomc* (7, 9, 18, 19). Riboprobes were generated from cDNA templates by in vitro transcription with a T3 (*ht2cr*) or SP6 (*Pomc*) polymerase, according to the manufacturer's protocol (Promega and Ambion Inc) in the presence of ^35^S-labeled UTP. cRNA riboprobes were diluted to 2 × 10^7^ cpm/mL in hybridization solution. Sections were mounted onto microscope slides, air-dried, and exposed to Biomax MR film (Kodak). Density of *ht2cr* mRNA and *Pomc* mRNA was examined in adjacent sections of brain tissue using densitometry analysis by computing the integrated density (the sum of the gray values minus background) using ImageJ (National Institutes of Health). This was performed at 3 ARC levels (−1.46, −1.82, and −2.30 mm from bregma) and the average integrated density was calculated.

### Calcium imaging

Coronal brain slices (180 μm) containing the ARC (−1.58 to −1.94 mm bregma) from male POMC-EGFP young adult (2.8 months ± 5 days, n = 4) and middle-aged (11.9 months ± 8 day, n = 4) mice were prepared as previously described (8) and maintained in an artificial cerebrospinal fluid solution containing (in mM) 126 NaCl, 2.5 KCl, 21.4 NaHCO_3_, 1.2 NaH_2_PO_4_, 10 glucose, 2 Na pyruvate, 1.2 MgCl_2_, 2.4 CaCl_2_ (pH 7.3), gassed with 95% O_2_ and 5% CO_2_. Slices were loaded with fura-2 AM (9.6μM, 0.04% pluronic acid, 34°C–36°C for 20 minutes, followed by 20 minutes washout). Imaging was performed at 34°C–36°C with an Olympus FluoView 1000MPE 2-photon laser-scanning microscope, equipped with a MaiTai DeepSee laser (Spectra-Physics) tuned to 790 nm and via an LUMPlanFI/IR ×40 (NA 0.8) objective. Images were acquired at a frame rate of 5.4 seconds, and spatial averages of somatic fluorescence were monitored as agonists were applied to the bath perfusion. Fluorescence values (F) were corrected for linear bleaching, background subtracted, and expressed as ΔF/F baseline.

### Drugs

5-HT reuptake inhibitor and releaser d-fenfluramine (Tocris Biosciences), 5-HT and NA reuptake inhibitor sibutramine (Tocris Biosciences), and 5-HT_2C_R agonists WAY-161,503 ([4aR]-8,9-dichloro-2,3,4,4a-tetrahydro-1H-pyrazino[1,2-a]quinoxalin-5(6H)-one; Tocris Biosciences) and lorcaserin (LGM Pharma) were used. Drugs were dissolved in 0.9% sterile saline and administered in vivo in a volume of 10 mL/kg body weight (ip).

### Data analysis

Data were analyzed with paired or independent *t* test, χ^2^, or repeated-measures ANOVA followed by Tukey's post hoc tests. For all analyses, significance was assigned at *P* < .05. Data are presented as mean ± SEM.

## Results

### 5-HT obesity medication appetite suppression requires ARC POMC signaling

**Figure 1.**
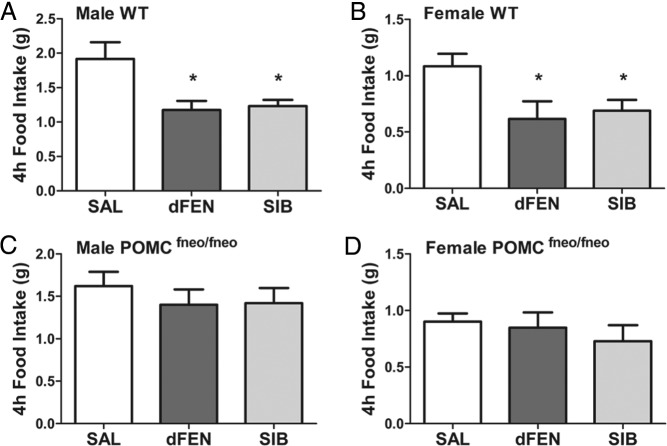
Selective ARC POMC deficiency prevents the anorectic effects of d-fenfluramine and sibutramine. A–D, Male (n = 6) (A) and female (n = 6) (B) wild-type mice (WT) significantly suppressed 4-hour dark cycle food intake after d-fenfluramine (dFEN) (3 mg/kg ip) and sibutramine (SIB) (5 mg/kg ip) treatment compared with saline treatment; in contrast, male (n = 6) (C) and female (n = 8) (D) littermates devoid of ARC POMC (POMC^fneo/fneo^) were not responsive to the anorectic effect of dFEN or SIB (repeated-measures ANOVA: main effect of treatment, *F*_2,44_= 11.51, *P* = .0001; main effect of sex, *F*_1,22_= 8.18, *P* = .0001; interaction between treatment and genotype, *F*_2,44_= 3.73, *P* = .032; no interaction between treatment and sex, *F*_2,44_= 0.79, *P* = .460; no interaction between genotype, treatment, and sex, *F*_2,44_= 0.317, *P* = .730; no main effect of genotype, *F*_1,22_= 0.089, *P* = .768). Importantly, these data confirm no difference by genotype in 4-hour food consumption; thereby, the genotypic difference is specific to the interaction between genotype and treatment. Specifically, WT and POMC^fneo/fneo^ littermates respond significantly differently to dFEN and SIB. As expected, male mice of both genotypes consumed a greater quantity of food compared with female littermates. *, *P* < .05 compared with saline analyzed with repeated-measures ANOVA followed by Tukey's post hoc comparisons.

We first sought to evaluate the contribution of ARC POMC to the appetitive effects of 5-HT obesity medications. Previous reports indicate that d-fenfluramine and 5-HT_2C_R agonists require melanocortin 4 receptors to produce effects on appetite (6, 7, 9); however, the role of the melanocortin system in sibutramine's appetitive effects has not been established. Although selective genetic manipulation of 5-HT_2C_R expression on POMC neurons illustrates the importance of the subpopulation of 5-HT_2C_R expressed with POMC in communicating 5-HT obesity drug effects (5, 10), these studies manipulate 5-HT_2C_R receptor expression, not POMC. Here we probe specifically whether ARC POMC neurons are required for the therapeutic effect of 5-HT obesity drugs d-fenfluramine and sibutramine.

d-Fenfluramine produced a significant reduction in food intake in male and female wild-type mice (Figure 1, A and B), but this effect was prevented in discrete ARC-deficient POMC^fneo/fneo^ littermates (Figure 1, C and D). Surprisingly, sibutramine, which blocks the reuptake of both 5-HT and NA, also required ARC POMC neurons to achieve its appetitive effects in male and female mice (Figure 1, A–D). Our results reveal a necessary mechanism through which obesity medications d-fenfluramine and sibutramine achieve their therapeutic appetitive effect, via activation of ARC POMC neurons.

### 5-HT compound anorectic potency is maintained with aging

**Figure 2.**
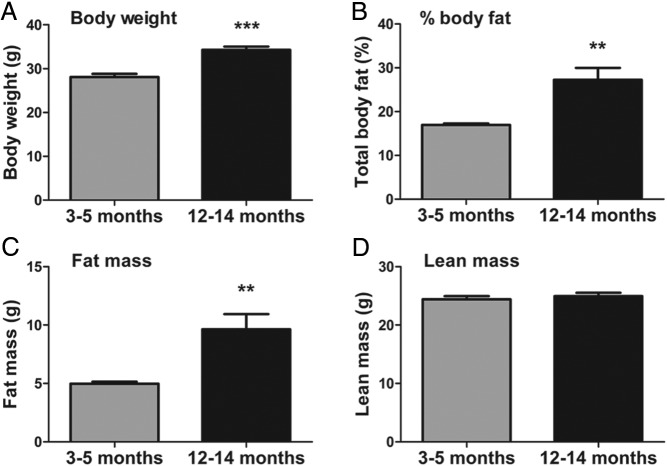
Middle-aged mice display an increase in body weight and fat mass. A–D, Body weight (*t*_10_ = 5.91, *P* = .001) (A), percent body fat (*t*_14_ = 3.35, *P* = .0054) (B), fat mass (*t*_14_ = 3.19, *P* = .0072) (C), and lean mass (*t*_14_ = 0.73, *P* = .49) (D) were assessed in young adult (3–5 months old, n = 7) and middle-aged (12–14 months old, n = 9) male C57BL/6 mice. Data are presented as mean ± SEM. **, *P* < .01; ***, *P* < .001 analyzed with independent-samples *t* test.

**Figure 3.**
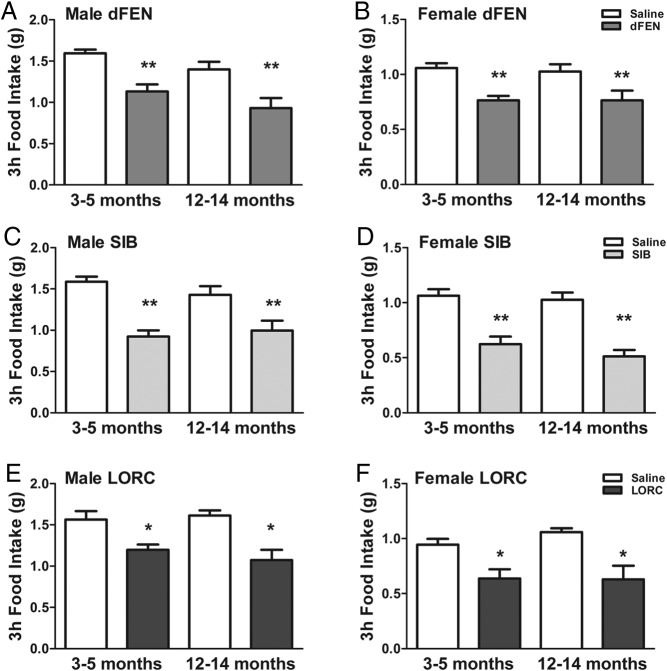
Lorcaserin, d-fenfluramine, and sibutramine anorectic potency is maintained with aging. A, C, and E, Three-hour dark cycle food intake in male mice aged 3–5 months (t_7_ = 4.97, *P* = .0018) or 12–14 months (t_7_ = 5.21, *P* = .0013) after d-fenfluramine (dFEN, 3 mg/kg) or saline (A), in male mice aged 3–5 months (*t*_7_ = 5.30, *P* = .0053) or 12–14 months (*t*_7_ = 5.00, *P* = .0088) after sibutramine (SIB, 5 mg/kg) or saline (C), or in male mice aged 3–5 months (*t*_4_ = 3.38, *P* = .028) or 12–14 months (*t*_7_ = 3.40, *P* = .012) after lorcaserin (LORC, 8 mg/kg) or saline (E). B, D, and F, Three-hour dark cycle food intake in female mice aged 3–5 months (*t*_7_ = 3.6, *P* = .0038) or 12–14 months (*t*_7_ = 3.91, *P* = .0082) after dFEN (3 mg/kg) or saline (B), in female mice aged 3–5 months (*t*_5_ = 5.34, *P* = .0075) or 12–14 months (*t*_5_ = 5.11, *P* = .0093) SIB (5 mg/kg) or saline (D), or in female mice aged 3–5 months (*t*_11_ = 3.00, *P* = .012) or 12–14 months (*t*_5_ =3.28, *P* = .022) after LORC (8 mg/kg) or saline (F). Data are presented as mean ± SEM. *, *P* < .05; **, *P* < .01 analyzed with paired *t test*.

Given that ARC POMC neuron function is diminished with age, we next sought to determine whether 5-HT compound anorectic potency is thereby compromised with aging. As expected, middle-aged (12–14 months old) male mice displayed significantly elevated body weight (Figure 2A), percent body fat (Figure 2B), and fat mass (Figure 2C), but not lean mass (Figure 2D), compared with young adult (3–5 months old) mice. However, d-fenfluramine (3 mg/kg), sibutramine (5 mg/kg), and lorcaserin (8 mg/kg) suppressed 3-hour dark cycle food intake to a comparable extent in young adult and middle-aged male (Figure 3, A, C, and E) and female (Figure 3, B, D, and F) POMC-EGFP mice. Food intake expressed as percent saline confirmed no differences in anorectic effect by age in mice treated with d-fenfluramine (male *t*_15_ = 0.73, *P* = .24, female *t*_9_ = 0.07, *P* = .47), sibutramine (male *t*_15_ = 0.45, *P* = .32, female *t*_9_ = 0.79, *P* = .22) or lorcaserin (male *t*_8_ = 1.22, *P* = .26, female *t*_10_ = 0.18, *P* = .86). These data indicate that diminished ARC POMC activity in middle-age does not impair 5-HT obesity therapy efficacy.

### Preserved ARC POMC and 5-HT_2C_R appetitive machinery with aging

**Figure 4.**
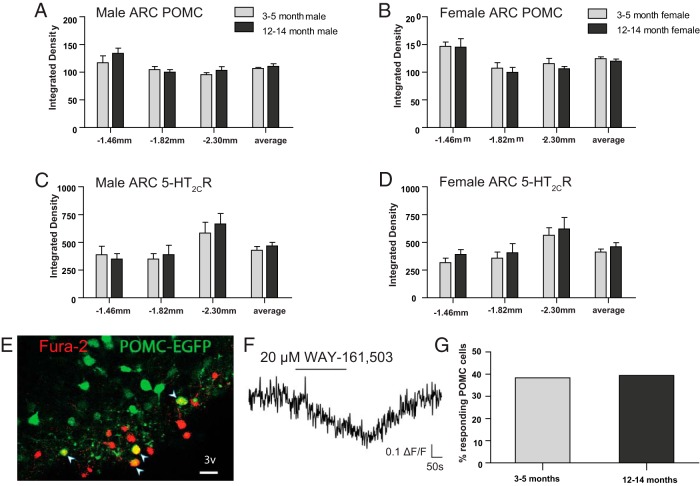
Preserved ARC 5-HT_2C_R–POMC appetitive machinery. A–D, No age-related differences in ARC gene expression were evident in male *Pomc* mRNA (t_3_ = 1.3, *P* = .27) (A) or *ht2cr* mRNA (t_3_ = 1.2, *P* = .31) (C) or female *Pomc* mRNA (t_3_ = 1.3, *P* = .51) (B) or *ht2cr* mRNA (t_3_ = 1.2, *P* = .29) (panel D) between young (3–5 months) and middle-aged (12–14 months) mice. E, A representative image of a coronal POMC-EGFP mouse brain slice illustrating EGFP neurons (green) loaded with fura-2 (red) in the ARC. All fura-2–loaded EGFP neurons (yellow, denoted with white arrow) were measured for changes in intracellular calcium, an assay of neuronal activation. Scale bar, 50 μm. F, Representative trace of POMC-EGFP neuron increasing intracellular calcium concentrations shown by decreasing fluorescence (F) expressed as ΔF/F to 20μM WAY-161503. G, 20 μM of 5-HT_2C_R agonist WAY161503 activates a similar proportion of ARC POMC neurons in young (23 from 60 cells) vs middle-aged (50 from 127) male EGFP brain slices using multiphoton fluorescence imaging to monitor changes in intracellular calcium (*X*^2^ (1) = 0.0184, *P* = .892). Data are presented as mean ± SEM, where appropriate. Abbreviation: 3v, third ventricle.

Given that 5-HT anorectic efficacy is maintained with aging, we surmised that it is POMC basal tone that is compromised with aging, not the 5-HT_2C_R and POMC neuroanatomical appetitive machinery. We therefore compared *Pomc* mRNA and *ht2cr* mRNA abundance in young adult (3–5 months old) and middle-aged (12–15 months old) male and female mice. Despite observing age-associated obesity, ARC *Pomc* mRNA (Figure 4, A and B) and *ht2cr* mRNA (Figure 4, C and D) did not vary with age in male and female mice. These data suggest that critical neural components for 5-HT appetite regulation at the level of gene expression are not reduced with age in parallel with weight gain.

To investigate the significance of this finding at the functional level, we determined whether pharmacological stimulation of the G_q_-coupled 5-HT_2C_Rs was equally effective in increasing ARC POMC cellular activity in brain slices from young adult and middle-aged POMC-EGFP mice. Using 2-photon fluorescence imaging to monitor changes in intracellular calcium, we observed that 5-HT_2C_R agonist WAY-161503 (20μM) activated a similar proportion of ARC POMC neurons in both young adult and middle-aged mice (Figure 4, E–G). Together, these data reveal that the density of POMC and 5-HT_2C_Rs expressed on an anatomical level is not altered with aging and, furthermore, that 5-HT_2C_R signaling within POMC neurons is not affected by age. This provides a mechanism through which 5-HT and 5-HT_2C_R agonist anorectic potency is maintained with age.

## Discussion

It is a clinical imperative to gain a greater understanding of the mechanisms underlying age-dependent obesity and, in turn, how these mechanisms impact the efficacy of obesity treatments. Like adult humans, adult mice accumulate adiposity with age, in the face of constant environmental conditions (ie, ad libitum access to the same diet and existing at a constant temperature). Although the biological factors driving increased fat accumulation with age are not well understood, recent reports reveal age-related melanocortin system remodeling and link these functional changes with age-associated obesity (13, 16). This age-related remodeled circuitry could thereby impact the efficacy of obesity medications requiring melanocortin system function to exert therapeutic effects.

Here we distill a critical and necessary mechanism responsible for communicating the anorectic effect of obesity medications d-fenfluramine and sibutramine to ARC POMC neurons. The mechanistic specificity of the hypophagic effect achieved via this small population of POMC neurons is remarkable given the wholesale effect on 5-HT and 5-HT/NA bioavailability produced by d-fenfluramine and sibutramine, respectively. Our findings are consistent with earlier reports demonstrating that 5-HT depolarizes and stimulates expression of ARC POMC neurons and that functional melanocortin 4 receptors (the primary appetitive receptor target of POMC) are required for d-fenfluramine and 5-HT_2C_R agonists to suppress food intake (7, 9, 20, 21). Subsequent work genetically manipulating 5-HT_2C_Rs expressed in global POMC neurons has emphasized the importance of this population of 5-HT_2C_Rs in 5-HT hypophagia. Specifically, d-fenfluramine and 5-HT_2C_R appetite suppression is attenuated in 5-HT_2C_R–null mice but fully restored in mice with 5-HT_2C_Rs exclusively re-expressed in POMC cells (5). These data are complemented by recent findings that selective inactivation of 5-HT_2C_Rs in POMC neurons abolishes d-fenfluramine and 5-HT_2C_R agonist hypophagia (10). Our results reveal a new level of regional specificity and furthermore indicate that independent of their expression of 5-HT_2C_Rs, POMC neurons specifically within the ARC are a critical component of the neural circuit through which obesity medications d-fenfluramine and sibutramine influence appetite.

Given that functional POMC is mechanistically required for 5-HT pharmacotherapy appetite suppression, recent reports revealing perturbed POMC function and melanocortin system remodeling in middle-age presented the possibility that 5-HT compound anorectic efficacy may be diminished with age. Specifically, middle-age is associated with an upregulation in mTOR signaling in ARC POMC cells, coupled with an age-dependent increase in inhibitory agouti-related protein inputs, which promotes lower resting membrane potential and reduction in POMC neuronal activity (13, 16).

Nevertheless, in the present study, we demonstrate that 5-HT compounds suppress feeding with equal potency in young adult and middle-aged mice. Furthermore, we report that the 5-HT_2C_R–POMC appetitive machinery is not compromised with middle-age; both ARC *ht2cr* and *Pomc* abundance is preserved, as is 5-HT_2C_R signaling in POMC cells. Specifically, plasma membrane G_q_-coupled 5-HT_2C_Rs activate phospholipase C, which hydrolyzes membrane phosphatidylinositol 4,5-bisphosphate into the second messengers inositol 1,4,5-trisphosphate and diacylglycerol, promoting calcium mobilization (3). Using 2-photon calcium imaging in the ARC of POMC-EGFP mice, we report that a 5-HT_2C_R agonist was equally effective in stimulating intracellular calcium mobilization in young and middle-aged mice, which suggests that there is no inherent defect in components of the intracellular signaling cascade lying downstream of the 5-HT_2C_R. Thereby, these findings provide a mechanism through which 5-HT medication anorectic potency is maintained with age and suggest that pharmacological activation of the 5-HT_2C_R-POMC system is sufficient to overcome any age-related reduction in endogenous POMC neuronal activity.

In summary, we report that 5-HT compound anorectic potency is maintained with aging in association with equivalent 5-HT medication activation of POMC neurons at comparable 5-HT_2C_R densities in the young adult and middle-aged brain. These data indicate that 5-HT compounds are capable of overcoming components of age-related changes in physiology to activate POMC neurons and suppress feeding. These data reveal that although former and current 5-HT obesity medications require ARC POMC to achieve appetitive effects, a pathway that is remodeled with aging, the anatomical 5-HT_2C_R-POMC machinery is preserved and the anorectic potency is maintained, findings of clinical significance to the global obese population.
